# Factors affecting health-related quality of life in Thai children with thalassemia

**DOI:** 10.1186/1471-2326-10-1

**Published:** 2010-01-21

**Authors:** Montarat Thavorncharoensap, Kitti Torcharus, Issarang Nuchprayoon, Arthorn Riewpaiboon, Kaemthong Indaratna, Bang-on Ubol

**Affiliations:** 1 Department of Pharmacy, Faculty of Pharmacy, Mahidol University, Bangkok, Thailand; 2 Department of Pediatrics, Phramongkutklao College of Medicine, Bangkok, Thailand; 3 Department of Pediatrics, Faculty of Medicine, Chulalongkorn University, Bangkok, Thailand; 4 Faculty of Economics, Chulalongkorn University, Bangkok, Thailand; 5 Department of Pediatrics, Saraburi Hospital, Saraburi Province, Thailand; 6 Health Intervention and Technology Assessment Program, Ministry of Public Health, Thailand

## Abstract

**Background:**

Knowledge of the factors associated with health-related quality of life (HRQOL) among patients with thalassemia is essential in developing more suitable clinical, counseling, and social support programs to improve treatment outcomes of these patients. In light of the limited research in this area, this study aims to examine factors associated with HRQOL among children and adolescents with thalassemia in Thailand.

**Methods:**

A cross-sectional survey was conducted in three selected hospitals in Thailand during June to November 2006. PedsQL™ 4.0 Generic Core Scale (Thai version) was used to assess HRQOL in 315 thalassemia patients between 5 and 18 years of age. Other related clinical characteristics of the patients were collected via medical record review.

**Results:**

The mean (SD) of the total summary score was 76.67 (11.40), while the means (SD) for the Physical Health Summary score and Psychosocial Health Summary score were 78.24 (14.77) and 75.54 (12.76), respectively. The school functioning subscale scored the lowest, with a mean of 67.89 (SD = 15.92). The following factors significantly affected the HRQOL of the patients: age; age at onset of anemia and age at first transfusion; pre-transfusion hemoglobin (Hb) level; receiving a blood transfusion during the previous three months; and disease severity. In addition, iron chelation therapy had a significant negative effect on HRQOL in the school functioning subscale. In contrast, serum ferritin level, frequency of blood transfusions per year, and gender were not significantly related to HRQOL among these patients. The results from multivariate analysis also confirmed these findings.

**Conclusions:**

To improve HRQOL of thalassemia patients, suitable programs aimed at providing psychosocial support and a link between the patient, school officials, the family and the physician are important, especially in terms of improving the school functioning score. The findings also confirmed the importance of maintaining a pre-transfusion Hb level of at least 9-10.5 g/dL. In addition, special care and attention should be given to patients with a severe condition, and those who are receiving subcutaneous iron chelation therapy.

## Background

Thalassemia is an inherited blood disease. It is a serious public health problem throughout the Mediterranean region, the Middle East and the Indian subcontinent, as well as in Southeast Asia [1]. Out of approximately 300 million carriers of this hemoglobin disorder worldwide [2], 55 million live in Southeast Asia [3]. In Thailand, with a population of 65 million, about 40% have thalassemia traits or are carriers, while 1% of the population is afflicted with this disease [4].

Thalassemia is a chronic disease that presents a range of serious clinical and psychological challenges. The effects of thalassemia on physical health can lead to physical deformity, growth retardation, and delayed puberty [5-7]. Its impact on physical appearance, e.g., bone deformities and short stature, also contributes to a poor self-image [6,7]. Severe complications such as heart failure, cardiac arrhythmia, liver disease, endocrine complications, and infections are common among thalassemia patients [8,9]. The problems mentioned do not only affect patients' physical functioning but also their emotional functioning, social functioning and school functioning, leading to impaired health-related quality of life (HRQOL) of the patients [6,7,10-14].

Although thalassemia is recognized as a genetic blood disorder which can be fatal if proper treatment is not received, during the past decades the development of new treatments and clinical management has markedly improved the prognosis as well as the survival rates of the patients [9,15-18]. However, the positive impact of these treatments is likely to be diminished, especially in terms of HRQOL, if they interfere with daily activities or are less tolerable. These may be the cases of regular blood transfusions that require frequent visits to the hospital, and nightly subcutaneous injections of iron chelation therapy [14].

For chronic diseases such as thalassemia, where a cure is not attainable and treatment may be prolonged, HRQOL is likely to be an essential outcome when considering options for treatment for individual patients and the allocation of health care resources. In child health service research, HRQOL is also recognized as an important health outcome [19-21]. As children are less able to voice their concerns and are more vulnerable than adults, the assessment of HRQOL in children is essential for the provision of proper care, since it helps in identifying the impacts of the disease and treatment from the children's perspective. Moreover, a recent study found that HRQOL of children can be used as a predictor of health care costs, and can also help identify at-risk groups of children who are candidates for proactive care coordination [22].

Due to the effects of the disease and its treatment, as mentioned previously, existing evidence indicates that thalassemia has a negative impact on HRQOL [6,11-13]. A previous study revealed that thalassemia patients experienced more depressive symptoms and reported lower HRQOL [6]. In addition, it was found that not only transfusion-dependent patients but also transfusion-independent patients suffered from impaired HRQOL [12]. By using EuroQoL (EQ-5D), a prior study [13] found that the pain domain of patients was affected the most, followed by the depression and mobility domains, with an equal score. One study [12] found that the most commonly affected domains were feelings such as depression, anxiety, and concern about overall health status; while another study [11] found that quality of life in the school domain was affected the most.

Presently, there is limited research examining the factors associated with HRQOL in children and adolescents with thalassemia. A previous study conducted in adults with thalassemia found that treatment and cultural difference did not affect HRQOL [7]. Other studies have found that age [11] and side effects of iron chelation [13,14] are predictors of HRQOL in children with thalassemia; while gender, ethnicity and household income are not [13]. A better understanding of the factors associated with HRQOL among children and adolescents with thalassemia could have a direct effect on the development of more suitable clinical, counseling, and social support programs to enhance treatment outcomes, especially in terms of HRQOL of these patients. In addition, at-risk groups of children and adolescents could be identified as candidates for proactive care assistance. Given the insufficient research in this area, this study aims to examine factors associated with HRQOL among thalassemic children and adolescents with the focus on patient perspective.

## Methods

### Participants and settings

The cross-sectional study was conducted among children and adolescents with thalassemia who received outpatient treatment at one of three participating hospitals - Phramongkutklao Hospital, King Chulalongkorn Memorial Hospital, and Saraburi Hospital - from June to November 2006. Phramongkutklao Hospital and King Chulalongkorn Memorial Hospital are teaching hospitals located in Bangkok, while Saraburi Hospital is a regional hospital located in Saraburi province (108 km from Bangkok). Participants in the study were selected based on the inclusion and exclusion criteria specified in Table 1. The demographic characteristics for the sample were later described in Table 2.

**Table 1:** Inclusion and exclusion criteria

Inclusion criteria	Exclusion criteria
Patients diagnosed with thalassemia.	Patients having impaired cognitive function or having severe clinical condition, which may limit their ability to participate in the study.
Patients between 5 and 18 years of age.	Patients taking oral iron overload treatment.
Patients who had been treated at Phramongkutklao Hospital, King Chulalongkorn Memorial Hospital or Saraburi Hospital since October 1, 2004.	Patients unwilling to participate in the study.

The sample size required for the study was calculated using the formula by Lemeshow et al. [23]. According to the formula, sample size should not be smaller than 30 times the total number of independent variables. Since the number of independent variables in this study is approximately 11, the total sample size required was 330.

### Study instruments

In this study, quality of life assessment was performed using the Pediatric Quality of Life Inventory™ (PedsQL™) 4.0 Generic Core Scale (Thai version). A user agreement was signed with the MAPI research Institute, Lyon, France, prior to use of the questionnaire. The PedsQL 4.0 Generic Core Scale includes parallel child self-reports (age ranges 5-7, 8-12 and 13-18 years) and parent proxy-reports (age ranges 2-4, 5-7, 8-12 and 13-18 years). PedsQL items ask how much of a problem a particular thing has been for patients during a certain period (the standard recall period is the past month). Item responses are measured on a five-point rating scale ranging from 0 (never a problem) to 4 (almost always a problem). The 23 items consist of 8 items on physical functioning, 5 items on emotional functioning, 5 items on social functioning and 5 items on school functioning, yielding a total score and two summary scores (i.e., Physical Health and Psychosocial Health). Each scale has a score ranging from 0-100, the higher score indicating higher QOL. The original version of PedsQL has demonstrated good internal consistency and validity in large samples of children with acute and chronic health conditions, as well as in samples of healthy children and adolescents [24-26]. Furthermore, previous use of the PedsQL 4.0 Generic Core Scale (Thai version) in Thailand indicated that it could distinguish between normal children and those with attention-deficit hyperactivity disorder (ADHD) [27].

Apart from the PedsQL questionnaire, the other instrument used in this study was a clinical record form. This form consisted of questions concerning demographics of the patients (e.g., gender, age, type of payment, hospital name, etc.), and clinical information (e.g., onset of anemia, diagnosis, age at first transfusion, history of blood transfusion, hemoglobin (Hb) level, complications, serum ferritin level, iron chelation treatment, etc.).

### Data collection

All eligible patients and their parents were approached as they came in for routine follow-ups at the thalassemia clinic in the Department of Pediatrics of each hospital during the data collection period. Written parental informed consent and the child's assent were obtained prior to participating in the study. At the beginning of the interview, all respondents were informed of the objectives of the study and were assured that all responses would remain confidential. For HRQOL assessment, the PedsQL questionnaires were to be completed independently by children with a minimum age of 8 years. Children aged 5-7 years were interviewed by trained interviewers. A clinical record form was then completed via medical record review for all patients in the study. The study was approved by the ethics committee of each participating hospital namely, Institutional Review Board of the Royal Thai army of Medical Department, Institutional Review Board of the Faculty of Medicine, Chulalongkorn University, and Institutional Review Board of Saraburi hospital.

### Data analysis

Data were analyzed by Microsoft Excel 2003 and SPSS (Statistical Package for the Social Sciences) program version 13.0. General characteristics of the patients were presented in terms of percentage, mean, and standard deviation. For HRQOL, both total HRQOL score and summary scores were presented in terms of mean and standard deviation. Pearson's correlation, chi-square, ANOVA, and T-test were used to examine the relationship between HRQOL and each demographic/clinical factor. Nonparametric tests were used if data were not normally distributed. Factors influencing the quality of life of children with thalassemia were later examined by multiple regression analysis.

Since a serum ferritin level above 2,500 ng/mL is associated with cardiovascular disease [15,16,28] and mortality [15], for data analysis serum ferritin level was classified into two levels: ≤2,500 ng/mL and > 2,500 ng/mL. With respect to pre-transfusion Hb level, pre-transfusion Hb of less than 7 g/dL indicates a need for regular blood transfusions. Also, pre-transfusion Hb level should be monitored routinely to maintain an optimal level of 9-10.5 g/dL [15,29]. As a result, in this study, pre-transfusion Hb was classified into three levels (i.e., <7 g/dL, 7-9 g/dL, and >9 g/Dl), In the classification of severity, the criteria used to determine severity in this study were pre-transfusion hemoglobin level, age at onset of anemia and age at first transfusion, and type of diagnosis. Since there was no consensus on classification of severity among thalassemia patients, for this study, the patient was classified as having a severe condition if any of the following applied: 1) his/her age at onset of anemia was less than 2 years, and age at first transfusion was less than 4 years; 2) a pre-transfusion hemoglobin level <7 g/dL; or 3) having been diagnosed with homozygous β-thalassemia (thalassemia major). Age at onset of anemia and age at first transfusion was used as one of the criteria, since a previous study [30] found it to be an important factor in predicting severity among thalassemia patients. In addition, Hb level was also used to classify severity of thalassemia in previous study [30]. Similarly, patient with homozygous β-thalassemia (thalassemia major) was also classified as severe thalassemia as they required chronic blood transfusion.

## Results

Demographic and clinical characteristic of the 315 thalassemic children and adolescents are presented in Table 2. It was found that about 51% of the participants were male. The percentages of patients diagnosed with β-thalassemia/HbE, homozygous β-thalassemia, and Hemoglobin H were about 53%, 5% and 42%, respectively. The mean age (SD) of the patients was 10 (3.3) years. About 42% of the patients had received a blood transfusion during the three months prior to the interview, while only 27% of them had received iron chelation therapy. Mean pre-transfusion Hb level (SD) measured three months prior to the HRQOL assessment was approximately 8.04 (1.2) g/dL, while mean serum ferritin level (SD) was approximately 2,510 (1,903) ng/mL. Only 6% of the patients reported having complications. Approximately 36% of the patients were classified as having a severe condition, as defined above.

**Table 2:** Patient characteristics

	N (%)/Mean (SD)
Gender (n = 314)	
Male	159 (50.6%)
Female	155 (49.4%)
Age (years) (n = 315)	10.0 (3.3)
Diagnosis (n = 310)	
β-thalassemia/Hb E	165 (53.2%)
Homozygous β-thalassemia	15 (4.8%)
Hemoglobin H	130 (42%)
Age at onset of anemia (years) (n = 270)	3.61 (2.6)
Age at first transfusion (years) (n = 191)	3.91 (2.8)
Receiving a blood transfusion during the previous 3 months (n = 245)	
Yes	103 (42%)
No	142 (58%)
Frequency of blood transfusion (n = 312)	
None	146 (46.8%)
Occasional (1-5 times/year)	58 (18.6%)
Low (6-12 times/year)	77 (24.7%)
High (13-22 times/year)	31 (9.9%)
Pre-transfusion Hb level 3 months prior to QOL assessment (g/dL) (n = 265)	8.04 (1.2)
Serum ferritin level (ng/mL) (n = 127)	2509.9(1903.6)
Iron chelation treatment (n = 314)*	
Yes	86 (27.4%)
No	228 (72.6%)
Complications (n = 314)	
Yes	19 (6.1%)
No	295 (93.9%)
Age at onset of anemia <2 and age at first transfusion <4 years old (n = 241)	
Yes	75 (31.1%)
No	166 (68.9%)
Having a severe condition** (n = 314)	
Yes	113 (36%)
No	201 (64%)

* Subcutaneous injection treatment

** Patients whose age at onset <2 and age at first transfusion <4 years, and/or patients diagnosed with homozygous beta-thalassemia, and/or patients with a pre-transfusion Hb level lower than 7 g/dL.

HRQOL scores based on child self-reports are presented in Table 3. Mean (SD) of the total summary score was 76.67 (11.40). When looking at the two summary scores, it was found that the means (SD) of the Physical Health summary score and Psychosocial Health summary score were 78.24 (14.77) and 75.54 (12.76), respectively. For the subscales of Psychosocial Health, the study revealed that school functioning scored the lowest (mean = 67.89; SD = 15.92), followed by emotional functioning (mean = 75.90; SD = 16.62) and social functioning (mean = 83.71; SD = 14.73).

**Table 3:** Quality of life scores of child self-report

Scale	Child self-report (n = 315)
	
	Mean ± SD
Total summary score	76.67 ± 11.40
Physical health	78.24 ± 14.77
Psychosocial health	75.54 ± 12.76
Emotional functioning	75.90 ± 16.62
Social functioning	83.71 ± 14.73
School functioning	67.89 ± 15.92

Table 4, 5, and 6 present HRQOL scores from the child self-report, classified by demographic, clinical, and treatment characteristics of the patients, respectively. As shown in table 4, age was found to be significantly associated with total summary score. The findings indicated that older patients were more likely to have significantly higher HRQOL compared to their younger counterparts. As displayed in table 4, the means of the HRQOL total summary scores (SD) for children aged 5-7, 8-12 and 13-18 years old were 73.91 (11.79), 75.51 (11.34) and 80.22 (10.44), respectively. On the other hand, gender was not associated with HRQOL scores.

**Table 4:** Quality of life scores of child self-report classified by demographic characteristics

		Child self-report					
		**Total summary score**	**Physical health**	**Psychosocial health**	**Emotional functioning**	**Social functioning**	**School functioning**
		
		**Mean (SD)**	**Mean (SD)**	**Mean (SD)**	**Mean (SD)**	**Mean (SD)**	**Mean (SD)**

Age: years (n = 315)							

5-7	n = 63	73.91(11.79)	74.80(16.57)	73.44(13.09)	73.17(19.24)	82.06(17.15)	65.08(16.35)

8-12	n = 153	75.51(11.34)	77.81(14.82)	74.17(12.57)	75.03(16.36)	81.70(14.77)	66.11(16.41)

13-18	n = 99	80.22(10.44)	81.09(12.96)	78.99(12.26)	78.99(16.36)	87.88(12.04)	72.42(13.97)

*P*-value		*0.001*	*0.054*	*0.003*	*0.138*	*0.003*	*0.003*

Gender (n = 314)							

Male	n = 159	78.87(11.31)	78.79(14.77)	75.86(11.15)	77.42(15.54)	83.58(13.63)	66.51(16.71)

Female	n = 155	76.42(11.54)	77.64(14.83)	75.11(14.24)	74.26(17.58)	83.81(15.85)	69.26(15.05)

*P*-value		*0.701*	*0.460*	*0.760*	*0.101*	*0.434*	*0.108*

**Table 5:** Quality of life scores of child self-report classified by clinical characteristics of the patients

		Child self-report					
		**Total summary score**	**Physical health**	**Psychosocial health**	**Emotional functioning**	**Social functioning**	**School functioning**
		
		**Mean (SD)**	**Mean (SD)**	**Mean (SD)**	**Mean (SD)**	**Mean (SD)**	**Mean (SD)**

Diagnosis (n = 310)							

β-thalassemia	n = 165	76.23(11.99)	77.46(13.20)	74.74(13.20)	76.03(17.36)	83.60(15.57)	67.06(14.92)

Homozygous β-thalassemia	n = 15	71.81(10.83)	73.54(13.91)	71.89(12.89)	70.00(16.58)	80.00(13.70)	62.67(15.68)

Hb H	n = 130	77.54(10.72)	79.49(14.68)	76.63(12.28)	76.23(15.76)	84.04(13.67)	69.23(17.19)

*P*-value		*0.145*	*0.187*	*0.234*	*0.368*	*0.424*	*0.103*

Age at onset of anemia <2 and age at first transfusion <4 years old (n = 241)							

Yes	n = 75	72.75(11.33)	73.96(15.95)	73.51(12.64)	74.53(14.71)	78.53(17.10)	63.26(14.94)

No	n = 166	77.72(11.08)	79.01(14.67)	76.02(12.73)	76.08(15.99)	85.49(13.45)	69.52(16.15)

*P*-value		*0.002*	*0.023*	*0.099*	*0.228*	*0.003*	*0.003*

Complications (n = 314)							

Yes	n = 19	77.52(14.37)	77.14(19.05)	77.81(16.21)	76.32(19.57)	84.21(16.52)	72.63(15.13)

No	n = 295	76.60(11.22)	78.30(14.50)	75.34(12.52)	75.83(16.46)	83.66(14.66)	67.56(15.97)

*P-value*		*0.773*	*0.941*	*0.169*	*0.628*	*0.661*	*0.228*

Having a severe condition* (n = 314)							

Yes	n = 113	74.07(12.05)	75.97(15.93)	74.12(12.77)	75.53(15.27)	78.89(16.86)	63.73(16.98)

No	n = 201	78.10(10.79)	79.49(15.93)	76.26(12.71)	76.04(17.37)	86.39(12.68)	69.63(15.09)

*P*-value		*0.004*	*0.077*	*0.097*	*0.417*	*<0.001*	*0.021*

Serum ferritin level (n = 127)							

<2500 ng/mL	n = 79	76.50(12.83)	78.20(15.66)	75.25(13.38)	74.18(18.70)	84.24(15.19)	66.27(14.82)

>2500 ng/mL	n = 48	76.61(10.14)	76.88(13.03)	75.76(12.73)	80.42(14.39)	84.06(13.94)	64.89(15.38)

*P*-value		*0.0897*	*0.426*	*0.903*	*0.100*	*0.726*	*0.741*

Pre-transfusion Hb level 3 months prior to QOL assessment (n = 265)							

<7 g/dL	n = 49	73.51(13.06)	75.06(17.84)	73.82(13.41)	74.03(14.21)	76.84(16.64)	64.90(18.78)

7-9 g/dL	n = 159	75.59(11.09)	77.10(14.61)	73.78(13.21)	87.01(13.09)	84.03(14.08)	66.00(15.55)

*>*9 g/Dl	n = 57	79.82(10.21)	81.03(13.35)	79.18(17.44)	76.49(17.44)	87.01(13.09)	74.03(14.21)

*P*-value		*0.012*	*0.163*	*0.016*	*0.474*	*0.002*	*0.008*

* Patients whose age at onset <2 and age at first transfusion <4 years, and/or patients diagnosed with homozygous β-thalassemia, and/or patients with a pre-transfusion Hb level lower than 7 g/dL

**Table 6:** Quality of life scores of child self-report classified by treatment characteristics of the patients

		Child self-report					
		
		Total summary score	Physical health	Psychosocial health	Emotional functioning	Social functioning	School functioning
		
		Mean (SD)	Mean (SD)	Mean (SD)	Mean (SD)	Mean (SD)	Mean (SD)
Receiving a blood transfusion during the previous 3 months (n = 245)							

Yes	n = 103	74.49(12.58)	75.00(16.19)	74.54(13.12)	74.85(18.37)	82.67(16.04)	65.15(16.90)

No	n = 142	78.51(10.55)	80.74(13.07)	76.84(12.53)	76.79(16.10)	85.35(13.18)	69.82(15.47)

*P*-value		*0.027*	*0.008*	*0.187*	*0.615*	*0.320*	*0.079*

Frequency of blood transfusion (n = 312)							

None	n = 146	78.05(10.77)	79.77(13.25)	76.76(12.53)	76.30(16.61)	85.68(13.55)	69.42(16.92)

Occasional	n = 58	76.52(10.86)	78.50(15.77)	74.37(12.40)	74.91(15.55)	82.33(15.59)	69.14(13.48)

Low	n = 77	75.06(11.97)	76.70(15.52)	74.13(13.78)	75.71(17.69)	81.16(15.47)	65.65(15.65)

High	n = 31	76.44(12.38)	76.91(16.00)	76.45(17.33)	76.45(17.33)	85.65(13.84)	66.45(14.21)

*P*-value		*0.42*	*0.62*	*0.45*	*0.72*	*0.13*	*0.26*

Iron chelation treatment* (n = 277)							

Yes	n = 61	74.39(12.24)	74.59(15.01)	76.64(13.75)	76.64(17.79)	82.62(15.04)	63.61(17.06)

No	n = 216	77.01(11.11)	78.73(14.85)	75.64(12.27)	75.21(16.34)	83.84(14.61)	69.21(15.67)

*P*-value		*0.214*	*0.051*	*0.759*	*0.343*	*0.601*	*0.026*

*subcutaneous injection treatment

Regarding clinical characteristics, having severe condition, pre-transfusion Hb level, age at onset of anemia and age at first transfusion were found to be significant predictors of total summary scores, as indicated in table 5. The study revealed that patients whose Hb level was higher than 9 g/dL had significantly higher total summary score (mean = 79.82; SD = 10.21) than those with Hb levels of 7-9 g/dL (mean = 75.59; SD = 11.09) or less than 7 g/dL (mean = 73.51; SD = 13.06). In addition, It was found that patients whose age at onset of anemia was <2 years and age at first transfusion <4 years had significantly low total summary scores. With regard to severity, it was found that the patients who were classified as having a severe condition had significantly low total summary score than their non-severe counterparts. On the other hand, diagnosis, complication, and serum ferritin level were not associated with HRQOL scores.

For treatment characteristics, receiving a blood transfusion during the previous three months was significantly associated with the total summary score, as shown in table 6. The study indicated that patients who had received a blood transfusion during the three months prior to HRQOL assessment had significantly lower total summary scores, as compared to those who did not receive a blood transfusion. On the other hand, frequency of blood transfusion was not associated with HRQOL scores.

When looking at each summary score, it was found that two factors - age at onset of anemia and age at first transfusion, and receiving a blood transfusion during the previous three months - were significantly related to the Physical Health summary score; while age and pre-transfusion Hb level were significantly related to the Psychosocial Health summary score. For the social and school functioning subscales, age, pre-transfusion Hb level, age at onset of anemia and age at first transfusion, and severity were significant predictors of HRQOL. In addition, iron chelation treatment was significantly associated with impaired HRQOL in the school functioning subscale.

Relationships between HRQOL score and patient characteristics were presented in Table 7, in terms of Pearson's correlation coefficient. Similar to what is shown in Table 4, 5, and 6, it was found that age and pre-transfusion Hb level were significant predictors of HRQOL, while serum ferritin level was not.

**Table 7:** Relationship between quality of life scores and patient characteristics by Pearson's correlation coefficient

	Pearson's correlation coefficient					
	
	Total summary score	Physical health	Psychosocial health	Emotional functioning	Social functioning	School functioning
Serum ferritin level (ng/mL)	-0.061	-0.091	-0.052	0.444	-0.043	-0.088

Pre-transfusion Hb level (g/dL)	0.170*	0.105	0.123**	0.009	0.222*	0.184*

Age (years)	0.250*	0.163*	0.185*	0.175*	0.217*	0.198*

* *p *< 0.05, ** *p *< 0.01

Table 8 presents the results of multivariate regression analysis in examining factors associated with the total summary score. It was shown that age and severity were significant predictors of HRQOL (i.e., total summary score). The findings revealed a positive relationship between age and HRQOL (β = 0.243, *p *< 0.001). Similarly, a significant relationship was also found between severity and HRQOL (β = -0.162, *p *= 0.003).

**Table 8:** Multivariate regression analysis results

	β	SE (β)	*P*-value
Constant	4.231	0.028	<0.001

Having a severe condition*	-0.162	0.017	0.003

Age (years)	0.243	0.003	<0.001

R^2 ^= 0.089, Y = Ln (total summary score)

* Patients whose age at onset <2 and age at first transfusion <4 years, and/or patients diagnosed with homozygous β-thalassemia, and/or patients with a pre-transfusion Hb level < 7 g/dL.

## Discussion

Since the focus of this study was on patient perspective then only HRQOL scores obtained from child self-report were examined. While data on parent proxy- report were also collected they will be the focus of subsequent manuscript and will be available upon request.

Similar to what was found in a previous study [11], the assessment of HRQOL of thalassemia patients in this study showed that psychosocial health had a lower score than physical health. Also, as with the previous study [11], the school functioning subscale scored the lowest. This could be explained by the fact that frequent absenteeism from school for hospital visits, and a lack of energy when performing academic activities, had a significant negative impact on the children's HRQOL [11,31-33].

Although the pattern of HRQOL scores was found to be the same as in a previous study [11], HRQOL scores obtained from this study were somewhat higher. Based on the previous study[11], the mean total summary score (SD), mean Physical health summary score (SD), and mean Psychosocial health summary score (SD) were 67.70 (12.40), 69.17 (15.34), and 67.39 (13.24), respectively. However, due to the absence of HRQOL scores of healthy children in Thailand as well as differences in country-specific characteristics, direct comparisons could be made with caution. Nevertheless, possible reason that could account for the difference in HRQOL scores between the two studies was that the patients in this study represented milder cases of thalassemia, as compared to those in the previous study [11]. About 60% of patients in the previous study were diagnosed with homozygous β-thalassemia, and 77% were transfusion-dependent. In contrast, only 4.8% of the patients in this study were diagnosed with homozygous β-thalassemia, and only 42% had received a blood transfusion during the previous three months.

Consistent with the findings from several studies conducted on other diseases [34-37], this study found that pre-transfusion Hb level and receiving a blood transfusion during the three months prior to HRQOL assessment were significant predictors of HRQOL among thalassemia patients. This could be explained by the fact that lower Hb level is associated with a number of symptoms, such as fatigue, general weakness, and decreased mental alertness, which might lead to impaired HRQOL of the patients in several domains. Similar to the recommendation for blood transfusion mentioned earlier - that pre-transfusion Hb level should be monitored routinely to maintain an optimal level of 9-10.5 g/dL [15,29] - the findings of this study indicated that patients with a pre-transfusion Hb level >9 g/dL had significantly higher HRQOL than patients whose pre-transfusion Hb level was <9 g/dL. In the case of blood transfusion, one possible explanation for the significant relationship between receiving a blood transfusion and lower HRQOL is that patients who received blood transfusions during the three months prior to HRQOL assessment were those with low pre-transfusion Hb levels. In contrast, this study found no significant relationship between frequency of blood transfusion per year and HRQOL. This non-significant relationship could be due to the fact that the questions used to assess HRQOL of the patients were related to the feelings and conditions of the patients during the previous month; therefore the number of transfusions per year might not be relevant to the HRQOL score.

Consistent with a previous study [11], age was a significant predictor of HRQOL among thalassemia patients. Although transitioning from adolescence to adulthood presents many new challenges - a more independent lifestyle, choosing a successful career path, and marriage [32] - this study found that adolescent patients had significantly higher HRQOL than their younger counterparts. This finding was similar to that of a previous study which revealed that older children with thalassemia experienced fewer symptoms of depression, reflecting a process of adjustment and coping [6].

Although existing evidence indicates that long-term iron overload might result in severe morbidity and mortality [15,16,28,38], and that a serum ferritin level higher than 2,500 ng/dL is associated with cardiac complications and mortality [15], this study found no relationship between serum ferritin level and HRQOL. This contradictory result could possibly be due to the fact that damaging, long-term iron overload occurs gradually; so short-term iron overload, as represented by an elevated serum ferritin level, did not cause significant visible symptoms or complications, and hence had no impact on their HRQOL. Similar to the findings of previous studies [13,14], this study found that, due to the burden of nightly subcutaneous injections of desferrioxamine 5 to 7 days per week [29], iron chelation treatment was significantly related to impaired HRQOL.

Complications and type of diagnosis were not related to HRQOL level in this study. This could be because the number of patients having complications was too small (only 6.1%) to detect a significant difference. Similarly, although patients diagnosed with homozygous β-thalassemia were expected to have significantly lower HRQOL since they were transfusion-dependent, a non-significant relationship between type of diagnosis and HRQOL was found in this study. This could possibly be due to the small number of patients diagnosed with homozygous β-thalassemia (< 5%) that were included in this study. Concordant with the findings from a previous study conducted on children with thalassemia [11], gender was not associated with HRQOL in this study.

One limitation of this study, as previously mentioned, is the absence of HRQOL scores of healthy children in Thailand. As a result, the true magnitude of thalassemia's impact on HRQOL was hardly estimated. Other limitation is that purposive sampling of the settings might limit the extent to which the results could be extrapolated to patients in other settings. In addition, failure to recruit adequate subjects, as stated in sample size calculation may be a potential limitation of the study. Lastly, as there was no consensus on how to determine severity of thalassemia patient, the criteria to assess severity of thalassemia varied across studies, ranging from only referred to patients with homozygous β thalassemia [39] to other different criteria [30,40,41]. As the result, the direct comparison across studies should be made with caution. The definition of severity used in this study was developed based on local expert opinions. As only data on type of diagnosis, hemoglobin level, age onset of anemia, and age at first transfusion were required to assess severity, this definition seemed to be feasible in less developed countries, where data on laboratory value or genetic information were unavailable in general practice.

## Conclusion

The findings of this study highlight the significant negative impact of thalassemia and its treatment on HRQOL in terms of physical functioning and psychosocial functioning, especially in the school functioning subscale. In light of this, and considering the prevalence of thalassemia in Thailand, our study suggested that modification of existing thalassemia management could be beneficial. Psychosocial and counseling programs aimed at helping patients discuss and accept their illness, facilitating a normal lifestyle, and providing a link between patients, school officials, the family and the physician may be helpful in alleviating these difficulties, especially academic performance. In addition, modification of health care services for children with thalassemia to become more patient-centered, flexible and comprehensive may reduce time spent at hospitals and also improve treatment outcomes, including HRQOL of the patients.

In the meantime, while stem cell transplantation is not considered as a standard treatment, blood transfusion and iron chelation therapy are required for thalassemia patients. The findings of this study confirmed the importance of maintaining pre-transfusion Hb level above 9 g/dL. Therefore, effective strategies aimed at promoting access to blood transfusions should be employed in several developing countries, where at present there is limited access to this essential treatment. In addition, given the fact that long-term iron overload leads to severe complications and increase mortality, a more convenient regimen of iron chelation that would prevent the long-term consequences of iron overload is essential and could help improve HRQOL of the patient [40-42], even in the situation where no significant relationship between the elevation of serum ferritin and HRQOL is found.

Furthermore, patients whose age at onset of anemia <2 years and age at first transfusion <4 years; those receiving blood transfusions whose pre-transfusion Hb <7 g/dL; those receiving iron chelation therapy; and those diagnosed with homozygous β-thalassemia deserve special attention and proactive care, since their HRQOL scores were relatively low.

## Competing interests

The authors declare that they have no competing interests.

## Authors' contributions

MT was involved in the study design; data collection, interpretation and analysis; and drafting the manuscript. KT, NI, AR were involved in the study design, data collection and interpretation, and revising the paper for intellectual content. BU was involved in the study design and data collection, and in revising the paper for important intellectual content. KI was involved in the study design and in revising the paper for important intellectual content. All authors read and approved the final manuscript.

## Pre-publication history

The pre-publication history for this paper can be accessed here:

http://www.biomedcentral.com/1471-2326/10/1/prepub
